# Liver Stiffness Using Transient Elastography is Applicable to Canines for Hepatic Disease Models

**DOI:** 10.1371/journal.pone.0041557

**Published:** 2012-07-20

**Authors:** Antonio Rivero-Juárez, Juan Morgaz, Angela Camacho, Pilar Muñoz-Rascón, Juan Manuel Dominguez, Raquel Sánchez-Céspedes, Julián Torre-Cisneros, Antonio Rivero

**Affiliations:** 1 Infectious Diseases Unit, Instituto Maimónides de Investigación Biomédica (IMIBIC), University Hospital Reina Sofía, Cordoba, Spain; 2 Department of Animal Medicine and Surgery, Veterinary School of Cordoba, Cordoba, Spain; 3 Department of Comparative Pathology, Veterinary School of Cordoba, Cordoba, Spain; Duke University, United States of America

## Abstract

**Background:**

The objectives of this study were to evaluate the best position and best exploration probe for determining liver stiffness (LS) in dogs using transient liver elastography (TE). Thirteen dogs were used in the study.

**Methodology/Principal Findings:**

Morphometric measurements taken were thoracic circumference, weight and height. Elastographic measurements were taken in 4 anatomical positions using two different probes: medium (M) and small (S). The exploration was considered correct when the success rate was above 60% and the interquartile range of the measurements did not exceed 30%. The best measurements were obtained in the middle of the 6th–9th intercostal spaces, with the dog in the left lateral position and using probe M for preference in adults and probe S mandatory for animals <2 years. The correlation between probes was 99%. Intra-observer variability showed an intra-class correlation of 97.6%.

**Conclusions/Significance:**

TE is a technique that is reproducible in dogs.

## Introduction

Chronic hepatitis C infection is a very common liver disease [1]. The outcome and prognosis of the disease are changing due to an improved knowledge of the pathogenesis of the infection, the discovery of new treatment targets and the optimization of patients monitored [2]–[5]. Animal models have played an important role in these challenges [6]–[9]. The main animal models designed for the study of HCV infection have been mouse-based [8]. These models however have several limitations, associated with immunological host factors and the fact that the mouse is not a natural host for HCV [10]–[11]. In this respect, a canine homolog of the human hepatitis C virus has recently been characterized [12]. This is the first step towards an HCV animal model not based on rodents for probing the pathogenesis, prevention and treatment of diseases caused by hepacivirus infection [a canine equivalent of the hepatitis C virus infection].

One of the most important parameters for optimization is the evaluation of liver damage and fibrosis stage. In this respect, transient liver elastography (TE) is a novel diagnostic method that enables the liver stiffness (LS) which is associated with liver fibrosis to be tested quickly and non-invasively. LS is directly related to tissue elasticity and its opposite, fibrosis. TE has a high sensitivity and specificity for diagnosing the presence or absence of significant fibrosis [13]–[21] and there is greater interobserver and intraobserver concordance [19]. Futhermore, TE explores a portion of liver tissue 100 times larger than that studied by liver biopsy [13]–[21]. In fact, TE is used quite extensively in clinical practice for patients affected with HCV, providing clinicians with a good deal of information about disease progression.

It may therefore be speculated that TE could be a way of monitoring experimental dogs chronically infected with hepacivirus, with considerable repercussions on future study models involving this canine equivalent of the hepatitis C virus. To date, however, the procedure has not been employed using dogs (bibliographic search). The objective of this study was to assess the reproducibility of the TE technique in dogs.

## Materials and Methods

### Ethics statement

This study was carried out in strict accordance with the recommendations in the Regulation for the Management of Laboratory of the Animals Federation of European Laboratory Animal Science (FELASA).

### Animals

Thirteen healthy dogs belonging to the Veterinary Teaching Hospital of the University of Cordoba were used in the study. The animals included in the study were beagles or greyhounds of between 1 and 7 years old, which showed no evidence of liver disease (defined as normal liver enzyme and bilirubin levels at the time of the study and 6 months previously), had not been included in other studies and had no concomitant diseases at the time of the study. The experimental protocol was approved by the Bioethics Committee of the University of Córdoba.

### Study Protocol

The thoracic circumference, weight and height of all animals were measured. We defined “overweight” as a body condition score of more than 6 [22].

LS measurements were taken using FibroScan (Echosens, Paris, France), an ultrasound transducer system, coupled to the axis of a vibrator that generates a vibration of low frequency and amplitude (50 Hz, 2 mm throw), causing elastic shear wave propagation through the tissue. The results of the measurements were expressed as kPa. Two probes were used to measure LS: a medium probe (M) used for adult humans, and a paediatric probe (S) with two modes: an S2 mode for dogs with a thoracic circumference wider than 45 cm, and S1 for dogs with a thoracic circumference not exceeding 45 cm.

A single observer trained in conducting transient elastography made the determinations (more than 200). The observer's general procedure in every instance was to carry out a sonographic examination using the same TE probe, in order to determine the best liver-tissue window in the various positions evaluated. When the best one was found, the elastography measure continued.

Four determinations were made of every animal in different anatomical positions in order to find the best one for carrying out an exploration. The anatomical positions studied were: position 1: lateral projection. On the right side, between the 6th and 9th intercostal spaces, with the animal in standing position; position 2: ventral projection exploration on the right side, just below the 8th rib, with the animal in standing position; position 3: lateral projection exploration in the 6th and 9th intercostal spaces on the right side, with the animal in left lateral position; position 4: ventral projection, between the xiphoid process and the 8th rib, with the animal in left lateral position. The LS of every animal was measured twice in each anatomical position, using the M or S probe according to circumference of the thorax, and with an interval of no less than 30 min between determinations in order to erase the skin impression made by the probe during the previous exploration. Determinations were made without sedation or anesthesia being used. Animals were shaved in the area of the anatomy being measured.

During each TE investigation, a minimum of 10 measurements were taken, with the mean of all of them established as the final value. An examination was considered suitable for analysis when 10 valid measurements had been taken, with a success rate (SR) of above 60% (SR60) for measurements, and when the interquartile range (IQR) between the 10 measurements was less than 30% (IQR30) [13]–[21].

An anatomical position was considered unsuitable for exploration by TLE if it did not meet the minimum quality criterion set (IQR30 and SR60) in more than 80% of animals explored. The position obtaining the highest SR and lowest IQR among the measurements was considered the best, and was selected as the reference position for determining LS in future studies.

### Statistical analysis

The SPSS statistical package, 18^th^ version (SPSS software, Chicago, Illinois, USA), was used to carry out statistical analyses. Categorical variables were evaluated using the Chi-square test. The intraclass correlation coefficient was used to evaluate concordance between the TE values taken with the two study probes and reproducibility.

## Results

The baseline characteristics of the population are shown in Table 1. The SR for determinations in position 1 was 53.8% (beagle SR 25%, greyhound SR 100%), while the SR in position 3 was 100% (100% for both breeds). At positions 2 and 4, and using both probes, no window was found in any dog of either breed that gave a successful determination of LS. Analysis of positions 1 and 3 by SR, IQR and LS values is shown in Table 2. Position 3 was considered the best one for determining LS in dogs.

**Table 1:** General characteristics of the population. Categorical variables are displayed as mean ± SD.

Characteristics	Beagle	Greyhound
N	8	5
Sex, male/female, n (%)	3 (37.5)/5 (62.5)	4 (80)/1 (20)
Weight (kg)	16.4±3.6	27.8±0.7
Thoracic circumference (cm)	60.3±4.7	64.4±1.5
Height (cm)	33.2±3.1	66.8±2.5
Age (years)	4.1±2.4	7.8±1.3
BCS≥6, n (%)	4 (50)	0

**Legend:** body composition system (BCS).

**Table 2:** Analysis of Liver Stiffness measurements in positions 1 and 3.

Code	Position 1						Position 3					
	LSM (kPa)	IQRM(kPa)	SRM (%)	LSS(kPa)	IQRS(kPa)	SRS(%)	LSM(kPa)	IQRM(kPa)	SRM(%)	LSS(kPa)	IQRS(kPa)	SRS(%)
**b1**	—	—	—	—	—	—	2.4	0.6	65	—	—	—
**b2**	3.8	1.2	54	—	—	—	3.2	0.9	78	2.9	0.5	80
**b3**	2.9	1	45	—	—	—	3.4	0.7	87	3.8	0.6	75
**b4**	—	—	—	—	—	—	3.6	1	80	3.2	0.8	95
**b5**	—	—	—	—	—	—	—	—	—	1.8	0.3	95
**b6**	—	—	—	—	—	—	2.4	0.5	70	2.2	0.4	85
**b7**	—	—	—	—	—	—	—	—	—	4	0.9	70
**b8**	—	—	—	—	—	—	—	—	—	3.4	0.9	75
**g1**	4.5	0.8	80	6.8	0.9	70	6.5	1.4	95	7.2	0.7	80
**g2**	6.8	1.1	74	7.1	0.5	75	5.8	0.9	90	6.3	0.9	97
**g3**	7.5	1.6	69	—	—	—	8.7	1.6	90	9.1	1.8	98
**g4**	6.5	1.2	67	6.8	1.6	70	6.2	0.8	90	6.8	1.6	94
**g5**	—	—	—	15.3	1.8	58	14.8	1.7	91	12.3	0.8	90

Legend: Beagle (b), Greyhound (g), Liver stiffness with probe M (LSM), Interquartile range with probe M (IQRM), Success rate with probe M (SRM), Liver stiffness with probe S (LSS), Interquartile range with probe S (IQRS), Success rate with probe S (SRS).

Using the M probe, there were no valid measurements for any dog under two years old, although probe S gave a valid measurement for all of them (p<0.001). The mean value of measurements was 5.2 kPa using the M probe, and 5.7 kPa with the S probe (*P* = 0.476). The mean value of results for beagles was 3.2 kPa using both probes (*P* = 0.999); for greyhounds, mean values were 6.5 kPa using the M probe and 7.5 kPa using the S probe (*P* = 0.704). The intraclass correlation coefficient between the probes was 98.7% for all determinations. Intra-observer reproducibility was 97.6%. Univariate analysis of possible success rate characteristics for determining liver stiffness is shows in Table 3.

**Table 3:** Univariate analysis of possible success rate characteristics for determining liver stiffness.

Characteristics	SRM (%)	SRS (%)	*P* value
**Age ≤2 years**	0	100	0.01
**Age >2 years**	100	80	
**Normal weight**	90	90	0.112
**Overweight**	33	100	
**Weight >15 kg**	75	100	0.204
**Weight ≤15 kg**	80	80	
**Height >33 cm**	75	100	0.361
**Height ≤33 cm**	80	80	
**TC ≤60 cm**	75	75	0.278
**TC >60 cm**	77	100	

**Legend:** Success rate with probe M (SRM), Success rate with probe S (SRS), thoracic circumference (TC).

## Discussion

TE is a new technique which is useful in human clinical practice for evaluating liver fibrosis sequentially and non-invasively [13]–[21], [23], [25]. In dogs, however, the technique is not standardized, and so has not so far been used with them. Our study is the first to evaluate the feasibility of this technique and shows that the tool may be applied to study models involving the canine equivalent of the hepatitis C virus.

Determining the proper position for scanning is the first step towards standardizing TE in dogs. To determine the best position and the best probe for exploration in each situation, we ranked the quality of the results obtained for each position, including the number and percentage of valid determinations and the IQR between determinations [15]–[18]. We used the same quality criteria in our study as those used in human studies, which show a good correlation between LS values and degree of liver fibrosis [15], [23], [24], [26]. According to the results of our study, the best position for TE in dogs is between the 6th and 9th intercostal spaces, with the animal in the left lateral position (position 3), since there was a 100% SR in our study population for explorations in this position. Our study found that TE applied between the 6th and 9th intercostal spaces on the right side, and with the animal in a standing position (position 1), yielded a lower SR. This position could be considered an alternative only in situations where it is not possible to use the reference position for some reason. The other positions tested (positions 2 and 4) did not give the minimum percentage of quality scans and should not therefore be considered for TE in dogs.

In our study, we used two probes to make determinations. We compared the determination values of the two probes in order to establish the correct probe for use, depending on the situation, with the aim of being able to explore a high proportion of patients in routine clinical practice. In our study, there was a 0% SR using probe M for dogs of less than two years old, although a determination could be made in every case using probe S. We found no relationship, in our study, between the morphometric parameters studied and the SR percentage, irrespective of the probe, although probe S was less useful than probe M for adults in terms of the group SR,. Therefore, our results suggest that, as with humans [26], the probe used to evaluate LS in dogs using TE should be adjusted to cater for the age of the animal.

On the other hand, there was very little variability obtained using the two probes. This phenomenon, as far as we know, has not yet been evaluated in humans. In our study there was a high correlation between the M and S exploratory probes and intra-observer reproducibility.

However, our study has several limitations. Firstly, the number of animals tested was low and studies using bigger populations are required. Secondly, LS was not compared with liver biopsy, and we could not therefore consider determination values or the presence or absence of liver disease. Studies testing this point in sick dogs are required are necessary and it should be addressed in further studies before TE enters canine clinical practice. Thirdly, our study only included two dog breeds and our results cannot therefore be extrapolated to other breeds. However, differences in TE technique are not expected in other breeds of similar morphometric characteristics. Fourthly, in our study, the LS values of the greyhounds were higher than those of the beagles. The reason for the different LS values between the two breeds is not known, although it could be due to different physiological and morphometric characteristics. For this reason, it might be hypothesized that normal LS values and cut-off levels for advanced fibrosis could vary depending on the breed. Studies evaluating the influence of breed on LS values are needed.

In conclusion, our study shows that TE is a reproducible technique in dogs, with the best results being obtained by exploring the animal between the 6th–9th intercostal spaces in the left lateral position. It seems that, as in human medicine, probe M should be used for adults and probe S for puppies. Therefore, the great utility of TE in the clinical practice of HCV infected patients could be extrapolated to models involving the canine equivalent of the hepatitis C virus.
